# Ultrasound of the Biceps Muscle in Idiopathic Parkinson’s Disease with Deep Brain Stimulation: Rigidity Can Be Quantified by Shear Wave Elastography

**DOI:** 10.3390/diagnostics13020213

**Published:** 2023-01-06

**Authors:** Julia Oppold, Maria-Sophie Breu, Alireza Gharabaghi, Alexander Grimm, Nicholas A. Del Grosso, Mohammad Hormozi, Benedict Kleiser, Philipp Klocke, Cornelius Kronlage, Daniel Weiß, Justus Marquetand

**Affiliations:** 1 Department of Epileptology, Hertie-Institute for Clinical Brain Research, University of Tübingen, 72076 Tübingen, Germany; 2 MEG-Center, University of Tübingen, 72076 Tübingen, Germany; 3 Department of Neurosurgery and Neurotechnology, Institute for Neuromodulation and Neurotechnology, University Hospital, University of Tübingen, 72076 Tübingen, Germany; 4 Centre for Neurology, Department of Neurodegenerative Diseases, and Hertie-Institute for Clinical Brain Research, University of Tübingen, 72076 Tübingen, Germany; 5 Department of Neural Dynamics and Magnetoencephalography, University of Tübingen, 72076 Tübingen, Germany

**Keywords:** Parkinson’s disease, rigidity, shear wave elastography, deep brain stimulation

## Abstract

Rigidity in Parkinson’s disease (PD) is assessed by clinical scales, mostly the Unified Parkinson’s Disease Rating Scale of the Movement Disorders Society (MDS-UPDRS). While the MDS-UPDRS-III ranges on an integer from 0 to 4, we investigated whether muscle ultrasound shear wave elastography (SWE) offers a refined assessment. Ten PD patients (five treated with deep brain stimulation (DBS) and levodopa, five with levodopa only) and ten healthy controls were included. Over a period of 80 min, both the SWE value and the item 22b-c of the MDS-UPDRS-III were measured at 5 min intervals. The measurements were performed bilaterally at the biceps brachii muscle (BB) and flexor digitorum profundus muscle in flexion and passive extension. Rigidity was modified and tracked under various therapeutic conditions (with and without medication/DBS). The feasibility of SWE for objective quantification was evaluated by correlation with the UPDRS-III: considering all positions and muscles, there was already a weak correlation (r = 0.01, *p* < 0.001)—in a targeted analysis, the BB in passive extension showed a markedly higher correlation (r = 0.494, *p* < 0.001). The application of dopaminergic medication and DBS resulted in statistically significant short-term changes in both clinical rigidity and SWE measurements in the BB (*p* < 0.001). We conclude that rigidity is reflected in the SWE measurements, indicating that SWE is a potential non-invasive quantitative assessment tool for PD.

## 1. Introduction

**Figure 1:**
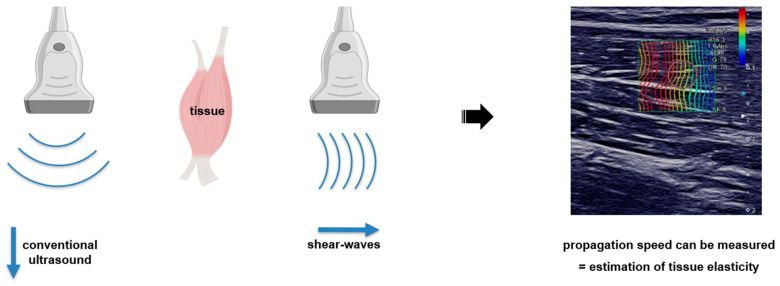
Visual description of muscle shear wave elastography (SWE). A conventional ultrasound propagates comparatively fast longitudinally through the tissue and provides a high image resolution (**left**), while a SW moves slowly transversely through the tissue and provides information about tissue elasticity in meters per second (**center**). On the **right**, is a screenshot of a Canon Aplio i800 system during sonography of the biceps brachii.

Shear-wave elastography (SWE) is an ultrasound imaging method for measuring tissue elasticity. Whereas in conventional B-mode ultrasound, sonic waves propagate with high speed longitudinally through a tissue, shear waves propagate transversely and comparably slower (Figure 1). The propagation speed of these shear waves offers an estimation of the underlying tissue elasticity, i.e., the faster the shear waves propagate through a tissue, the stiffer it is. While SWE is the modality, the measured parameter is usually expressed as shear wave velocity (SWV) in meters per second (m/s) or, using a conversion formula, as Young’s modulus (Pascal) [1].

SWE is already an established diagnostic tool in the staging of liver cirrhosis [2] and thyroid [3] or breast lesions [4]. In the case of liver cirrhosis, the stiffness corresponds to the progression of the fibrotic remodeling and the disease course (e.g., the higher a liver’s SWE measurement, the more advanced the stage of cirrhosis). Recent research is also focusing on functional changes as in the case of increased muscle tone in skeletal muscle tissue [5,6,7]; here, the diagnostic yield of SWE is still under investigation.

Especially in neurological disorders leading to a functionally increased muscle tone such as Parkinson’s disease (PD), SWE might be of diagnostic utility. In PD, along with bradykinesia and tremor, rigidity is one of the cardinal symptoms. So far, the evaluation of rigidity in PD has been based on clinical examination. The assessment can be standardized, for instance, according to the Unified Parkinson’s Disease Rating Scale of the Movement Disorder Society (MDS-UPDRS) [8]. The inter-rater variability between experts and trained staff for UPDRS-III is generally high (ICC = 0.749, *p* < 0.0001) [9,10], but in everyday clinical practice under time pressure, objective parameters are sought. The UPDRS achieves a high test–retest reliability with an ICC of 0.9 [9], indicating a high intra-rater reliability [10,11,12]. However, UPDRS only allows a coarse-grained assessment within an integer from 0 to 4, whereas SWE offers a more fine-grained evaluation.

The main reason to include DBS patients aside from medication-only patients is that one of the often-encountered difficulties in routine DBS outpatient department visits is that attempts to optimize the stimulation parameters still consist of empirically chosen stimulation adjustments that oftentimes rely on the clinicians’ experience and the observation of an immediate clinical change [13,14]. For these visits, DBS patients are not usually deprived of their medication, and so any clinical change can be the results of parameter adjustments. While greater parameter changes my lead to a more macroscopic clinical change, more subtle changes in amplitude or pulse width may not automatically be ascertained by the human eye or touch. Using shear wave elastography as a control measure could deliver objective and, more importantly, instantaneous assurance about the clinical efficacy of the currently set DBS parameters. Behavioral clinical objective outcome measures may also integrate parameter optimization in other movement disorders, e.g., SWE was already used to test the cervical muscles in 30 patients with torsional cervical dystonia. SWE has diagnostic value as it provides significantly higher (*p* < 0.01) values for the more affected side and the healthy control (*p* < 0.01) [15].

Several studies have assessed SWE at a group level on different muscles in PD patients. Du et al. [16] applied SWE to PD patients and found increased stiffness of the biceps brachii (BB) compared to healthy controls. Ding et al. [17] examined the BB and brachioradialis muscle (BR) and collected the related UPDRS-III values; they discovered a positive correlation (BR r = 0.344, *p* = 0.22; BB r = 0.419, *p* = 0.005) between the SWE measurements and the UPDRS-III values. Yin et al. [18] performed a study on the gastrocnemius muscle during passive extension in PD patients at different ankle angles and described a negative correlation (r = −0.719, *p* < 0.001) between SWV and the slack angle.

Whereas the previous studies were cross-sectional, the present work explored the utility of SWE for the longitudinal assessment of rigidity in PD patients, aiming to track short-term therapeutic effects over a time course of 80 min. This study aimed to show how rigidity changes, by therapeutic intervention, are reflected by SWE. To the best of our knowledge, this is also the first application of SWE in PD patients treated with deep brain stimulation (DBS).

## 2. Materials and Methods

### 2.1. Subjects

A total of 10 patients with PD exhibiting the akinetic–rigid or mixed features fulfilled the criteria of idiopathic PD (Table 1) and were recruited from the Department of Neurology, University Hospital Tübingen, Tübingen, Germany, from May to September 2021 (age: 69.1 ± 7.7 [54–78] years). The patients came to our department regularly, so diagnosis verification benefited from a long-term monitoring. The diagnosis of IPS and its subtypes was based on history and clinical neurological examination [19]. The criteria for this were those described by the UK Parkinson’s Disease Society Brain Bank [10]. All patients received dopaminergic medication, with 5 patients being treated additionally with DBS. In an initial exploratory phase, some tremor-dominant PD patients were studied, but it was decided not to include them in the study because tremor severely affected the SWE measurements. For comparison, 10 healthy control subjects (HC) were examined. Matching by age and sex was omitted as in comparable SWE studies [20], among other reasons because the primary aim of this study was to show a longitudinal effect within PD, not to compare the results of PD patients to those of HC.

All participants were over the age of 18 and provided informed consent. The ethics committee of the University of Tübingen approved this study (project number 770/2020BO2), and all examinations were carried out according to the Declaration of Helsinki.

### 2.2. Study Protocol

A Canon Aplio i800 system with a 14 MHz linear transducer (i14LX5/PLI-120BX, Canon Medical System, Neuss, Germany) was used to measure SWV. First, the biceps brachii (BB) and flexor digitorum profundus (FDP) muscles were identified by B-mode ultrasound, and a homogeneous area was selected. The ultrasound probe was then positioned in longitudinal alignment to the muscle fibers with the least possible amount of pressure applied, as proposed by Wang et al. [22]. SWV was subsequently measured at a depth of two to four centimeters. For standardization purposes and to maintain the same measurement position for each measurement, the ultrasound probe placement area was marked with patch strips on the skin. The following device settings were chosen: the frame rate was adjusted to 5, smoothing and time smoothing were set to the maximum. SWV was measured in m/s.

**Figure 2:**
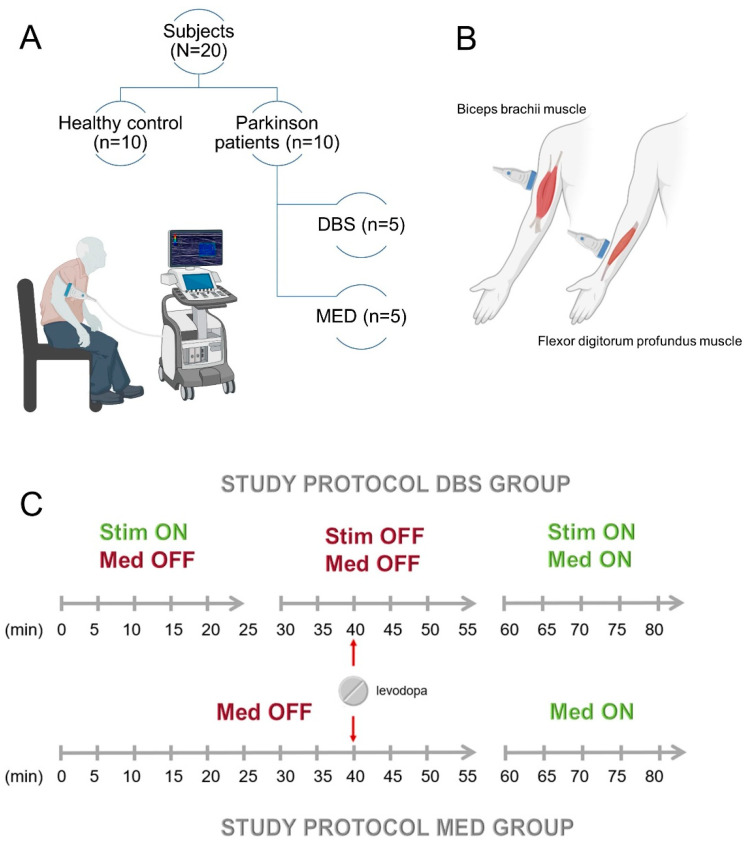
(**A**) The figure depicts the measurement setup; the patients were comfortably seated on a chair while the upper extremity was examined sonographically. The subject collective consisted of a total of 10 healthy controls and 10 Parkinson’s disease patients; of the latter, 5 were treated with deep brain stimulation (DBS), and 5 with medication (MED). (**B**) The biceps brachii muscle and flexor digitorum muscle were measured. (**C**) Illustration of the experimental protocol of the longitudinal measurement over 80 min. Shear wave velocity and the Unified Parkinson’s Disease Score were assessed every five minutes. Rigidity was modulated: at minute 40, the regular medication containing levodopa with benserazide or carbidopa was administered as a tablet (MED ON) to both parallel groups. In the DBS group, stimulation was additionally switched off between minutes 30 and 55 (STIM OFF).

All subjects (N = 20) were seated comfortably in a chair (Figure 2A), and SWV of the BB and FDP were measured bilaterally in positions of both flexion and maximum passive extension. Therefore, a subject’s elbow was in either of two positions (1) flexed approximately 90 degrees with the forearm supinated (‘flexed position’) and (2) with the arm in full passive extension, the forearms supinated, and the dorsal elbow facing downwards (‘maximum passive extension position’).

Over a total period of 80 min, SWV and item 22 of the MDS-UPDRS-III scale were collected at 5 min intervals, resulting in 15 measurements on 4 muscles each (Figure 2C). The item 22b-c of the MDS-UPDRS-III scale testing rigidity during passive movements of the large joints (ranging from 0 to 4) was collected on the upper limb bilaterally at the wrist and elbow joints. The assessment was performed unblinded in this pilot study because of the exploratory design and due to the short time intervals. The rater was always the same trained medical student (JO) in advanced educational stage who had already chosen a focus on neurology and had extensively familiarized with the method through hands-on training and supervision. Rigidity was either decreased or increased by turning DBS on (STIM ON) or off (STIM OFF), respectively.

A regular evening intake of tablets containing levodopa (levodopa, 50–200 mg with benserazide, 12.5–50 mg or carbidopa, 25 mg) was incorporated into the study design. By maintaining the regular therapeutic regimen, the patients were optimally therapied and were neither rigid nor hyperkinetic.

In our a priori hypothesis, this resulted in 3 time intervals for the DBS patients:

- DBS ON/MED OFF—medium rigidity
- DBS OFF/MED OFF—maximal rigidity
- DBS ON/MED ON—minimal rigidity

For the MED patients, there were two phases:

- MED OFF—maximal rigidity
- MED ON—minimal rigidity

This proof-of-principle study’s primary hypothesis was that SWE may objectively quantify rigidity in PD, previously altered by therapeutic interventions (MED ON/OFF, STIM ON/OFF). To support this hypothesis, the acquired data, SWE and UPDRS were correlated in order to compare the well-established UPDRS with SWE and propose the latter as a potentially finer assessment tool. Secondarily, insights were obtained on the ideal measurement point consisting of muscle and position by selecting the ones with the highest correlation. This acquired information will in return help further SWE studies optimize their protocols. The longitudinal acquisition of UPDRS under different therapeutic interventions and their effects were analyzed and compared between subject groups.

### 2.3. Statistical Analyses

Statistical analyses were performed using SPSS Statistics Premium (version 28, IBM, Armonk, NY, USA) and JMP (SAS, Cary, NC, USA). The images were created using the BioRender (https://biorender.com/, accessed on 23 February 2022). Descriptive statistics with mean ± standard deviation (SD) was calculated for the SWV measurement results of BB and FDP in different positions separately for all three subject groups (MED, DBS, HC). In addition, quartiles were calculated due to the non-normal distribution and small sample size. Because normality could not be assumed, the SWE variables between the subject groups were compared using the nonparametric Kruskal–Wallis test. For the ordinal MDS-UPDRS-III (‘22b-c’) variables of MED and DBS groups, the Mann–Whitney U-test was used. The time intervals I-III (I: DBS ON/MED OFF, II: DBS OFF/MED OFF, III: DBS ON/MED ON) were compared using the Friedman test and Bonferroni-corrected post hoc tests. Spearman Rho correlation was chosen to correlate MDS-UPDRS-III (‘22b-c’) with SWE. The significance level was set at *p* < 0.05.

## 3. Results

### 3.1. SWV Varies among the Muscles, Positions, and Participant Groups

The mean SWVs over 80 min are listed in Table 2. They were significantly higher (*p* < 0.001) in PD patients (all PD Patients 2.5 ± 0.8 [1.2–6.4] m/s, DBS 2.5 ± 0.7 [1.2–4.9] m/s and MED 2.4 ± 0.9 [1.3–6.4] m/s) than in healthy controls (2.2 ± 0.5 [1.4–3.8] m/s). In addition, differences by position, muscle, or participant group were consistently observed. The comparison with the respective side (right or left) showed no significant differences (*p* = 0.28). In flexion, the mean SWV was significantly lower (1.9 ± 0.5 (1.2–4.6) m/s) than in extension (2.9 ± 0.6 (1.8–6.4) m/s) (*p* < 0.001), and the BB (2.5 ± 0.9 (1.3–6.4) m/s) was slightly stiffer than the FDP (2.3 ± 0.6 (1.2–4.8) m/s) (*p* < 0.001).

### 3.2. SWV and MDS-UPDRS-III (‘22b-c’) Show a Positive Correlation

**Figure 3:**
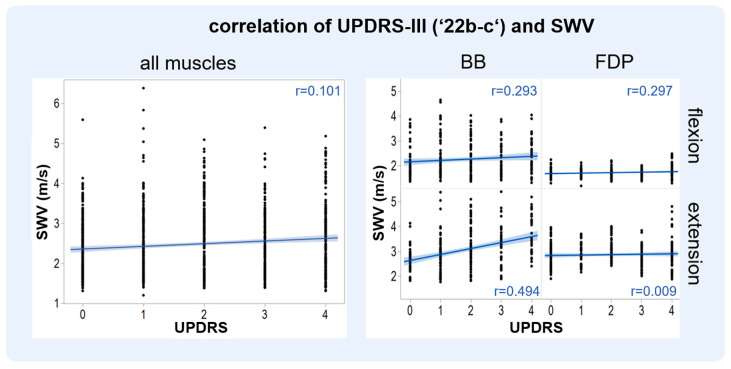
(**Left**): Overall Spearman’s Rho correlation (r) analysis of the Movement Disorder Society Unified Parkinson’s Disease Rating Scale (MDS-UPDRS) with shear wave velocity (SWV) for all Parkinson’s patients and muscles (biceps brachii and flexor digitorum profundus muscle). A significant positive correlation (r = 0.1, *p* < 0.001) between MDS-UPDRS III item 22b–c and SWE can be seen. (**Right**): Spearman’s Rho correlation (r) analysis of MDS-UPDRS with SWV for all Parkinson’s patients. Correlations were divided into muscle groups (biceps brachii and flexor digitorum profundus) and position (extension or flexion). A correlation of r = 0.494, *p* < 0.001 was seen for the biceps muscle in extension.

Spearman’s Rho correlation analysis showed a significant positive correlation between the item 22b-c of MDS-UPDRS-III and the respective muscle SWV (Table 3, Figure 3). Overall, the SWV of the four examined muscles correlated weakly with the MDS-UPDRS-III item 22b-c (r = 0.1, *p* < 0,001). Analysis of individual muscles showed that the SWV varied per muscle and position. The BB in extension showed the highest correlation (r = 0.494; *p* < 0.001); therefore, further analysis was performed during different therapeutic conditions with the BB in extension.

### 3.3. SWV and MDS-UPDRS-III (‘22b-c’) of the BB Alter under Varying Therapeutic Conditions

**Figure 4:**
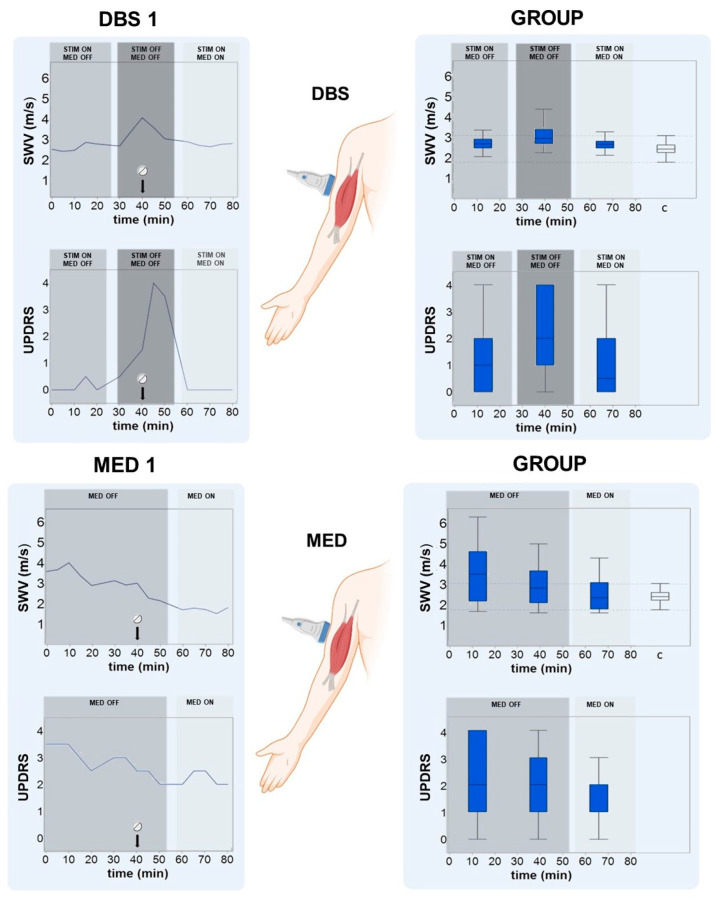
Shear wave velocity (SWV) and Movement Disorder Society Unified Parkinson’s Disease Rating Scale (MDS-UPDRS) in the biceps brachii (BB) muscle in passive extension in deep brain stimulation (DBS) and medication-only patients. The SWV and MDS-UPDRS-III item 22b-c were recorded in 5 min intervals longitudinally over a total period of 80 min. DBS was turned on and off (DBS ON/OFF). Regular levodopa with benserazide or carbidopa was timed to the 40th minute of the measurement to include the therapeutic effect of levodopa (MED ON/OFF). (**Left**): individual measurements of one exemplary patient with DBS (DBS1). (**Right**): group comparisons. It becomes evident that SWV and MDS-UPDRS rose and fell with various therapeutic conditions and correlated positively. The comparisons between different timepoints were statistically significant (*p* < 0.001). Boxplots on the far right labeled with ‘c´, with SWE measurements from healthy controls as a reference, were included.

Since the BB in extension showed the highest correlation (r = 0.494), the measurements in this muscle were used for individual analyses and comparisons of altering therapeutic conditions. Significant differences (*p* < 0.001) between the different therapeutic conditions (time intervals 1, 2, and 3) were observed. Figure 4 shows boxplots of the BB in extension with SWV and MDS-UPDRS ratings, of MED and DBS patients, as well as exemplary individual patients’ plots.

### 3.4. The SWV Are Consistent with Previous Works

Previously published SWE studies on PD also examined BB in extension. A comparison can be found in Table 4.

## 4. Discussion

This proof-of-principle study demonstrated that changes in rigidity in PD patients are reflected by objective changes in SWE, underlining the potential of SWE as an imaging tool for rigidity in PD patients. Intake of 100 mg/25 mg of levodopa/benserazide and, in some patients, switching DBS on and off induced changes in rigidity. The muscle SWV correlated significantly (overall: r = 0.1, *p* < 0.001) with the clinical standard for quantifying rigidity (MDS-UPDRS-III item 22b-c).

### 4.1. Choice of Muscle, Technical Considerations for SWE

In PD patients, the highest correlation coefficient between SWV and clinical rigidity (quantified by MDS-UPDRS-III item 22b-c) was found for the BB in passive extension (r = 0.494, *p* < 0.001). The BB therefore seems to be particularly suitable, a conclusion shared by Romano et al. [5]: in fact, a higher variance was found for the FDP (3.07 ± 1.57 m/s; 2.3 ± 0.37 m/s) than for the BB (4.14 ± 0.97 m/s; 1.95 ± 0.3 m/s). This could be explained by the anatomical structure of the BB with a comparably low pennation angle [5], so that the muscle fibers are measured in a true longitudinal section [5]. Another factor that could explain the superiority of the BB over the FDP is that it is more superficial and therefore easier to measure in B-mode. In addition, Alfuraih et al. [23] demonstrated that the variance of SWE increases with depth, which speaks in favor of measuring the BB. In general, the region of interest in this study was mostly placed at a 2 cm depth, but never deeper than 4 cm.

To obtain the most possible valid values, further technical considerations were made: besides pennation angle, joint position and measurement depth, also the pressure applied to the probe as well as the amount of ultrasound gel and the patient BMI were considered [23,24]. The BMI of patients and HC was similar at 25.3 ± 5.3 (18.8–33.2) kg/m^2^; other possibly confounding factors were also considered and adjusted comparably (Table 1).

### 4.2. Comparison to SWE in Post-Stroke Spasticity

Increased muscle tone is found not only in PD but also in spastic post-stroke rigidity. Whereas PD corresponds to an extrapyramidal lesion with increased resistance in all directions of movements, pyramidal spasticity is associated with increased resistance in the initial movement and in specific directions. Despite differences in etiology and resulting muscle remodeling, it is interesting to investigate how SWE is valid for other diseases with increased muscle stiffness, to possibly use it across neuromuscular diseases. In ischemic and hemorrhagic stroke patients, Wu et al. [25] examined the BB of the paretic arm and the healthy half of the body with SWE. The paretic side (90° flexion: 2.23 ± 0.15 m/s, extension: 3.28 ± 0.11 m/s) was stiffer than the healthy side (90° flexion: 1.88 ± 0.08 m/s, *p* = 0.002, extension: 2.93 ± 0.06 m/s, *p* < 0.002). This goes along with our increased SWE values in extension compared to flexion; in addition, it suggests that SWV is applicable to other pathologic states of increased muscle tone.

### 4.3. Comparison to Other Studies of SWE in PD

In PD, SWV measurements of the BB in extension were also performed by Ding et al. [17] (3.65 ± 0.46 m/s) and Du et al. [16] (3.99 ± 2.83 m/s; 4.28 ± 2.64 m/s). The measured values are comparable to those obtained in this study, though up to 1 m/s higher. This could be due to various biases, e.g., different ultrasound equipment and probes, level of pressure applied, therapeutic conditions, etc. (Table 4). In contrast to the previous studies, here, rigidity was (a) examined longitudinally over 80 min and (b) under different therapeutic conditions (with and without DBS/medication), and (c) MDS-UPDRS-III (‘22b-c’) was collected and correlated at the same 5 min intervals.

### 4.4. Other Technical Means and Relevance of the Rigidity Assessment in PD

Mechanical and electromyography-based measurements of rigidity have been shown to reflect similar short-term therapeutic effects in a similar set up in animal experiments: Mera et al. [26] applied a custom-designed automated arm rigidity testing system in three female rhesus monkeys with induced Parkinsonism and DBS. The measurements were taken over 60 min during flexion and extension of the elbow. Rigidity decreased by up to 70% after DBS was turned on and by up to 90.5% after apomorphine administration, similar to our study.

In humans with DBS, accelerometers and EMG were used to assess stiffness intraoperatively during nine DBS implantations to optimize the search for the best electrode location [27]: the number of potential stimulation sites increased with the accelerometer from 144 to 170 of 188 possible sites examined. According to Journee et al. [28], accelerometers were preferable to EMG because of the low signal-to-noise ratio. In a systematic review, Ferreira-Sánchez et al. [8] described the good validity and correlation of clinically assessed rigidity by servomotors and other biomechanical sensors. However, these methods are not yet established in clinical practice. SWE could be a possible method to assess rigidity in such settings. Prior to this, a comparison of the above-mentioned methods in a larger study is required.

### 4.5. Limitations

This study has the following limitations. (1) The measurement conditions (elbow joint angle, ultrasound probe placement) were kept constant as best as manually possible; however, a better technical solution such as a probe holder would have been potentially better. (2) The studies regarding the effect of age on SWE are ambiguous. Some reported increased SWV with age [29,30], while some reported a decreased value [24,31,32]. More recent studies [5,20] have led to the assumption that the differences regarding age and sex could remain negligible within the respective SD, though those clinical characteristics should be taken more into account in an extended, larger study. (3) We only correlated the SWV with clinical assessments according to the MDS-UPDRS. The use of other modalities (e.g., accelerometer, force measurement, surface EMG) in parallel may be considered to underline the validity of our results. (4) Part of the study was the artificial modification of rigidity by STIM ON/OFF, MED ON/OFF. The timing of medication and DBS effects was not precise: in some patients, the effects of DBS started immediately, in others after 20 min or later. The time course of washout for axial symptoms may be longer; however, a longer washout period would have rendered the study setup unfeasible for a routine outpatient’s department visit and thus reduce its clinical applicability. The modulation of rigidity was more pronounced in the DBS group compared to the MED group. In the MED group, pharmacological aspects, e.g., absorption of levodopa, gastroparesis, and protein composition, play a role. It may be insightful to examine patients who have been off medication overnight or who are treated with an intrajejunal levodopa pump. In addition, concomitant medication was not considered, e.g., MAO inhibitors and dopamine agonists. (5) The measurement duration should be shortened because the elderly PD patients were quickly exhausted, especially in the MED OFF/DBS OFF phase.

## 5. Conclusions

SWE measurements represent a potential novel non-invasive biomarker for rigidity. Here, we provide the proof of principle that SWE can monitor the short-term effects of dopaminergic medication and DBS in PD patients. Possible clinical applications include optimizing drug dosages or stimulation parameters and verifying the DBS electrodes’ placement.

## Figures and Tables

**Table 1:** Baseline characteristics of the Parkinson’s patients.

ID	Age (yrs)	Gender	BMI (kg/m^2^)	Hoehn and Yahr	MDS- UPDRS III		LEDD, mg	Type	More Affected Side	Disease Duration, (yrs)	Time with DBS, (yrs)
					On	Off					
DBS1	73	F	23.7	3	39	52	963	ET	L	21	13
DBS2	73	F	33.2	2.5	12	34	595	AR	E	10	1
DBS3	71	F	22.5	2.5	16	40	415	ET	L	16	1
DBS4	66	M	22.8	3	39	53	979	AR	L	12	4
DBS5	78	M	21.7	4	32	83	1650	AR	R	17	4
MED1	75	M	32.7	2	10	41	1200	AR	R	11	n/a
MED2	58	M	23.4	3	n/a	n/a	730	ET	L	13	n/a
MED3	74	M	22.1	4	43	68	325	ET	R	2	n/a
MED4	69	M	18.8	2	25	47	563	ET	R	13	n/a
MED5	54	M	32.1	3	11	24	1656	ET	L	7	n/a

Abbreviations: BMI = body mass index, LEDD = levodopa equivalent daily dose calculated according to Tomlinson et al., [21], MDS-UPDRS III = Movement Disorder Society Unified Parkinson Disease Rating Scale III, F = female, M = male, ET = mixed type, AR = akinetic–rigid type, L = left, R = right, E = equivalent. n/a = not applicable. DBS = deep brain stimulation. MED = medication-only group. For MED 2, MDS-UPDRS III evaluation is missing due to incomplete data entry.

**Table 2:** Mean shear wave velocity (SWV) indicated for the groups of participants in different muscles and positions over an 80 min time interval. Mean ± standard deviation, quartiles 25/50/50 (minimum—maximum). Overall average values of SWV in meters per second [m/s] for both muscles biceps brachii (BB) and flexor digitorum profundus (FDP), in two positions: Flexion and extension.

	HC	All PD Patients (MED + DBS)	HC vs. MED + DBS	MED	DBS
Mean SWV [m/s]	2.2 ± 0.5 (1.4–3.8) 1.7/2.2/2.7	2.5 ± 0.8 (1.2–6.4) 1.8/2.4/3	*p* < 0.001	2.4 ± 0.9 (1.3–6.4) 1.8/2.2/2.9	2.5 ± 0.7 (1.2–4.9) 1.8/2.6/3
SWV of BB in flexion [m/s]	1.8 ± 0.4 (1.4–3.0) 1.6/1.7/1.9	2.3 ± 0.7 (1.3–4.7) 1.7/2/2.7	*p* < 0.001	2.1 ± 0.6 (1.3–4.6) 1.6/1.9/2.4	2.4 ± 0.7 (1.4–4.0) 1.8/2.2/3.1
SWV of BB in extension [m/s]	2.6 ± 0.3 (1.9–3.2) 2.4/2.5/2.7	3.1 ± 0.9 (1.8–6.4) 2.5/2.9/3.4	*p* < 0.001	3.1 ± 1.1 (1.8–6.4) 2.1/3/4	3.0 ± 0.5 (2.2–4.9) 2.7/2.9/3.1
SWV of FDP in flexion [m/s]	1.7 ± 0.1 (1.5–2.2) 1.7/1.7/1.8	1.7 ± 0.2 (1.2–2.5) 1.6/1.7/1.8	*p* = 0.078	1.7 ± 0.2 (1.3–2.4) 1.6/1.7/1.8	1.7 ± 0.2 (1.2–2.5) 1.6/1.7/1.8
SWV of FDP in extension [m/s]	2.8 ± 0.3 (2.0–3.8) 2.6/2.8/3	2.9 ± 0.5 (1.9–4.8) 2.5/2.8/3.1	*p* = 0.083	2.7 ± 0.5 (1.9–4.8) 2.3/2.6/3	3.0 ± 0.4 (2.0–4.1) 2.8/3/3.3

Abbreviations: DBS = deep brain stimulation. MED = medication-only group. HC = healthy control. MED + DBS = all patients with Parkinson’s disease.

**Table 3:** Spearman’s Rho correlation (r) between MDS-UPDRS-III (‘22b-c’) and shear wave velocity (SWV) over 80 min for all patients (therapy with and without DBS), broken down by muscle group and position (r), showing a positive correlation of the UPDRS with SWV. This trend is most evident for the biceps brachii (BB) in extension.

	All PD Patients (MED + DBS)
BB in flexion	r = 0.294; *p* < 0.001
BB in extension	r = 0.494; *p* < 0.001
FDP in flexion	r = 0.297; *p* < 0.001
FDP in extension	r = 0.009; *p* = 0.91

BB = Biceps brachii. FDP = flexor digitorum profundus. DBS = deep brain stimulation. MED = medication-only group.

**Table 4:** Summary of muscle SWE reference values (SWV in m/s) sorted by the authors.

	This Study	Ding et al. (2021) [17]	Du et al. (2016) [16]
Therapeutic condition	Off and On	On	Off
Cohort	N = 10	N = 63	N = 46
SWE of BB in extension in m/s	DBS: 2.7 ± 0.69 MED: 2.62 ± 1.03	3.65 ± 0.46	3.99 ± 2.83 4.28 ± 2.64

Abbreviations: Mean ± standard deviation DBS = deep brain stimulation. MED = medication-only group.

## Data Availability

Raw data will be made available upon reasonable request.
